# DCGAN-DTA: Predicting drug-target binding affinity with deep convolutional generative adversarial networks

**DOI:** 10.1186/s12864-024-10326-x

**Published:** 2024-05-09

**Authors:** Mahmood Kalemati, Mojtaba Zamani Emani, Somayyeh Koohi

**Affiliations:** Department of Computer Engineering, Sharif University of Technology, Tehran, Iran

**Keywords:** Drug-target binding affinity, Deep convolutional generative adversarial networks, BLOSUM encoding, Adversarial control experiments, Straw models

## Abstract

**Background:**

In recent years, there has been a growing interest in utilizing computational approaches to predict drug-target binding affinity, aiming to expedite the early drug discovery process. To address the limitations of experimental methods, such as cost and time, several machine learning-based techniques have been developed. However, these methods encounter certain challenges, including the limited availability of training data, reliance on human intervention for feature selection and engineering, and a lack of validation approaches for robust evaluation in real-life applications.

**Results:**

To mitigate these limitations, in this study, we propose a method for drug-target binding affinity prediction based on deep convolutional generative adversarial networks. Additionally, we conducted a series of validation experiments and implemented adversarial control experiments using straw models. These experiments serve to demonstrate the robustness and efficacy of our predictive models. We conducted a comprehensive evaluation of our method by comparing it to baselines and state-of-the-art methods. Two recently updated datasets, namely the BindingDB and PDBBind, were used for this purpose. Our findings indicate that our method outperforms the alternative methods in terms of three performance measures when using warm-start data splitting settings. Moreover, when considering physiochemical-based cold-start data splitting settings, our method demonstrates superior predictive performance, particularly in terms of the concordance index.

**Conclusion:**

The results of our study affirm the practical value of our method and its superiority over alternative approaches in predicting drug-target binding affinity across multiple validation sets. This highlights the potential of our approach in accelerating drug repurposing efforts, facilitating novel drug discovery, and ultimately enhancing disease treatment. The data and source code for this study were deposited in the GitHub repository, https://github.com/mojtabaze7/DCGAN-DTA. Furthermore, the web server for our method is accessible at https://dcgan.shinyapps.io/bindingaffinity/.

**Supplementary Information:**

The online version contains supplementary material available at 10.1186/s12864-024-10326-x.

## Background

The prediction of binding affinity between candidate drugs and target proteins, which can impact disease conditions, is a key early stage in the drug discovery and development pipeline [1, 2]. However, experimental methods such as immunoprecipitation, spectroscopy, calorimetry, and surface plasmon resonance have been developed to characterize binding affinity [3–8]. However, these approaches often depend on limited structural information from drug-target pairs, specialized domain knowledge, and expensive and time-consuming experimental assays [2, 9].

To address these challenges, computational approaches have been developed that utilize available protein amino acid sequences and compound SMILES. These approaches aim to predict drug-target binding affinity (DTA) quickly and cost-effectively, overcoming the scarcity of structural information and the need for domain expert knowledge [10–26]. By leveraging computational methods, DTA prediction becomes more accessible and efficient, facilitating exploration of potential drug-target interactions and aiding in drug discovery [27].

There are several computational approaches for DTA prediction, especially machine learning (ML) and deep learning (DL) based methods [10–26]. These methods can be categorized into four groups: similarity-based, sequence-based, graph-based, and transformer-based methods. They utilize protein sequences and drug SMILES to extract meaningful features for the prediction of DTA.

KronRLS [10] and SimBoost [11] are two prominent similarity-based methods that rely on Smith-Waterman [28] and PubChem [29] similarities for proteins and drugs, respectively. NTFRDF [15], which addresses the prediction task as a classification problem, focuses on capturing topological differences and utilizes a multi-similarity fusion strategy to enrich network features. These methods employ machine learning techniques to predict binding affinities and interactions by constructing matrices that capture the complex relationships between drug-target pairs, based on their respective similarities. While these methods demonstrate significant prediction performance, they face challenges in feature selection and engineering from the available protein sequences and drug SMILES data. This limitation can potentially impact the predictive accuracy and robustness of these methods.

To overcome the limitations of similarity-based ML methods, sequence-based approaches that, such as DeepDTA [16], have been developed. This method leverages the raw protein sequences and drug SMILES, encoding them and feeding them into a CNN-based network for automatic feature extraction. While sequence-based methods that utilize deep neural networks have shown promising performance in predicting DTA, they often rely on a limited amount of labeled data for effective feature extraction. In order to tackle this challenge, GANsDTA [17] introduces a semi-supervised framework for DTA prediction using generative adversarial networks [30]. By incorporating unlabeled data for feature extraction, this method aims to improve performance. However, GANsDTA’s use of the fully connected-based GANs may not adequately capture the local patterns present in protein sequences and drug SMILES, resulting in only marginal improvements compared to previous approaches.

Graph-based methods leverage graph neural networks (GNNs) [31–33] for representation learning by constructing graph-like representations for proteins and drugs. For instance, GraphDTA [18] represents drugs using graphs and employs GNNs to learn features from the graph representation of drugs. Dynamic graph DTA (DGDTA) [19] utilizes a dynamic graph attention network to assess the significance of drug features. It is coupled with a bidirectional long short-term memory (Bi-LSTM) network to capture contextual information from protein sequences. GPCNDTA [20] constructs drug graphs based on their 2D topology and physicochemical properties, and protein graphs based on their contact matrix and the physicochemical properties of residues. It employs a residual CensNet and a residual EW-GCN to extract features from drugs and proteins. While molecular graphs offer rich structural information about drugs, the use of GNNs requires an additional step for modeling the graph and converting from drug SMILES, resulting in increased computational overhead in terms of time and space complexity.

With advancements in transformer architecture [34], the application of transformers for feature extraction from protein sequences and drug SMILES has gained prominence [35, 36]. One notable method in this domain is FusionDTA [23], which introduces a transformer-based network in combination with a LSTM network for drug and protein feature extraction. By leveraging transformers and LSTMs, FusionDTA captures long-term dependencies and aims to learn a distributed representation for drugs and proteins. MRBDTA [24] introduces the Trans block, which improves the transformer’s encoder and incorporates skip connections at the encoder level to enhance the extraction of molecule features and the capability to identify interaction sites between proteins and drugs. TEFDTA [25] introduced an attention-based transformer encoder. This model utilizes converted drug SMILES to MACCS fingerprints to capture substructure information of drugs, enabling the prediction of binding affinity values for drug–target interactions. G-K BertDTA [26] utilized knowledge-based BERT (KB-BERT) to capture semantic features of SMILES molecules, in conjunction with a graph isomorphism network (GIN) to learn relational features between isomorphic SMILES structures. While transformers and LSTMs offer valuable capabilities in capturing long-term dependencies and learning distributed representations, the time and space complexity associated with these methods must be carefully managed, particularly as the dataset size increases.

In summary, many DTA prediction methods encounter challenges such as human intervention in feature selection and engineering, the need for additional computational tools for modeling, reliance on complex and computationally intensive models, and a lack of available labeled data for effective representation learning. Moreover, the validation of these methods often relies on limited experiments, which may not adequately assess the impact of confounding variables and experimental artifacts, potentially leading to overoptimistic prediction performance. Therefore, it is crucial to consider DTA methods that address these challenges and limitations by leveraging the advantages of deep neural networks, such as their ability to automatically learn features from raw data and handle complex relationships.

In this paper, we propose a method called DCGAN-DTA, which is a deep CNN-based generative adversarial network for drug-target binding affinity prediction. The architecture of DCGAN-DTA consists of a DCGAN-based network [37] for extracting features from the protein sequences and drug SMILES using unlabeled data. This is followed by a CNN-based network for local feature extraction from the sequences and a fully-connected network for DTA prediction. The use of CNNs and fully connected-based GANs for protein and drug representation learning in DTA prediction has been previously demonstrated in [16, 17]. Additionally, the effectiveness of DCGANs in medical applications has been showcased in various studies [38, 39]. Motivated by these studies and aiming to harness the capabilities of one-dimensional CNNs for learning sequence patterns in protein sequences and drug SMILES data, as well as leveraging CNN-based generative models for efficient feature extraction from unlabeled data in large databases, we explored the utilization of a customized version of deep generative adversarial networks for DTA prediction. Building on this foundation, we propose the application of DCGANs for DTA prediction. DCGAN-DTA employs a four-step process for drug-target binding affinity (DTA) prediction. Firstly, encoding and embedding techniques are applied to drug SMILES and protein sequences. Secondly, a customized version of deep convolutional generative adversarial networks (DCGANs) is utilized for feature extraction. Notably, DCGAN-DTA diverges from traditional DCGANs in architecture and activation functions to suit sequence data dynamics. Thirdly, an add layer is employed for merging latent vectors, enhancing model performance compared to concatenation layers. Finally, a fully-connected block is utilized for prediction.

To evaluate the performance of our sequence-based method, we conducted multiple experiments using two recently updated datasets, BindingDB [40] and PDBBind [41]. We also employed physiochemical splitting data strategies to assess the generalization and robustness of our DTA method. Furthermore, we conducted adversarial control experiments using straw models [42] to validate the prediction performance and generalization of our method.

In summary, the key contributions of DCGAN-DTA can be outlined as follows:

- Proposal of utilizing a customized one-dimensional deep convolutional generative adversarial network (DCGAN) for feature extraction from drug SMILES and protein sequences.
- Incorporating evolutionary features from proteins through BLOSUM encoding and employing the Add layer for feature fusion in a generative AI-based model for drug-target binding affinity prediction, aiming to enhance prediction performance and reduce network complexity.
- Enable prediction based on data-splitting settings for the physicochemical properties of compound molecules.
- Provision of robust prediction performance through purposeful adversarial control experiments.

## Method

### Datasets

We conducted our evaluation using two well-known and recently updated datasets: BindingDB and PDBBind. The BindingDB dataset contains binding affinities measured by Inhibition constant (Ki), Dissociation constant (Kd), and IC50 [40]. For our evaluation, we specifically utilized the Kd version of the BindingDB dataset due to its larger number of compounds and proteins. After refining the dataset according to recommended guidelines for data harmonization and stable training, we obtained a dataset comprising logarithmic-transformed binding affinities (pKd) for 9864 small molecules and 1088 protein targets.

Regarding the PDBBind dataset, we selected the refined version (v2020), which includes binding affinity data of higher quality compared to the general and core sets [41]. To ensure data consistency, we excluded redundancies arising from multiple sequences for the same drugs. The refined set consists of logarithmic-transformed Ki and Kd binding values for 4231 compound SMILES and 1606 protein sequences.

Supplementary Table 1 provides a summary of the benchmark datasets, including the number of protein sequences, drug SMILES, and drug-target interactions. Further details regarding the drug SMILES, protein sequences, and binding affinities of both datasets can be found in Supplementary Fig. 1.

### DCGAN-DTA method

**Fig. 1:**
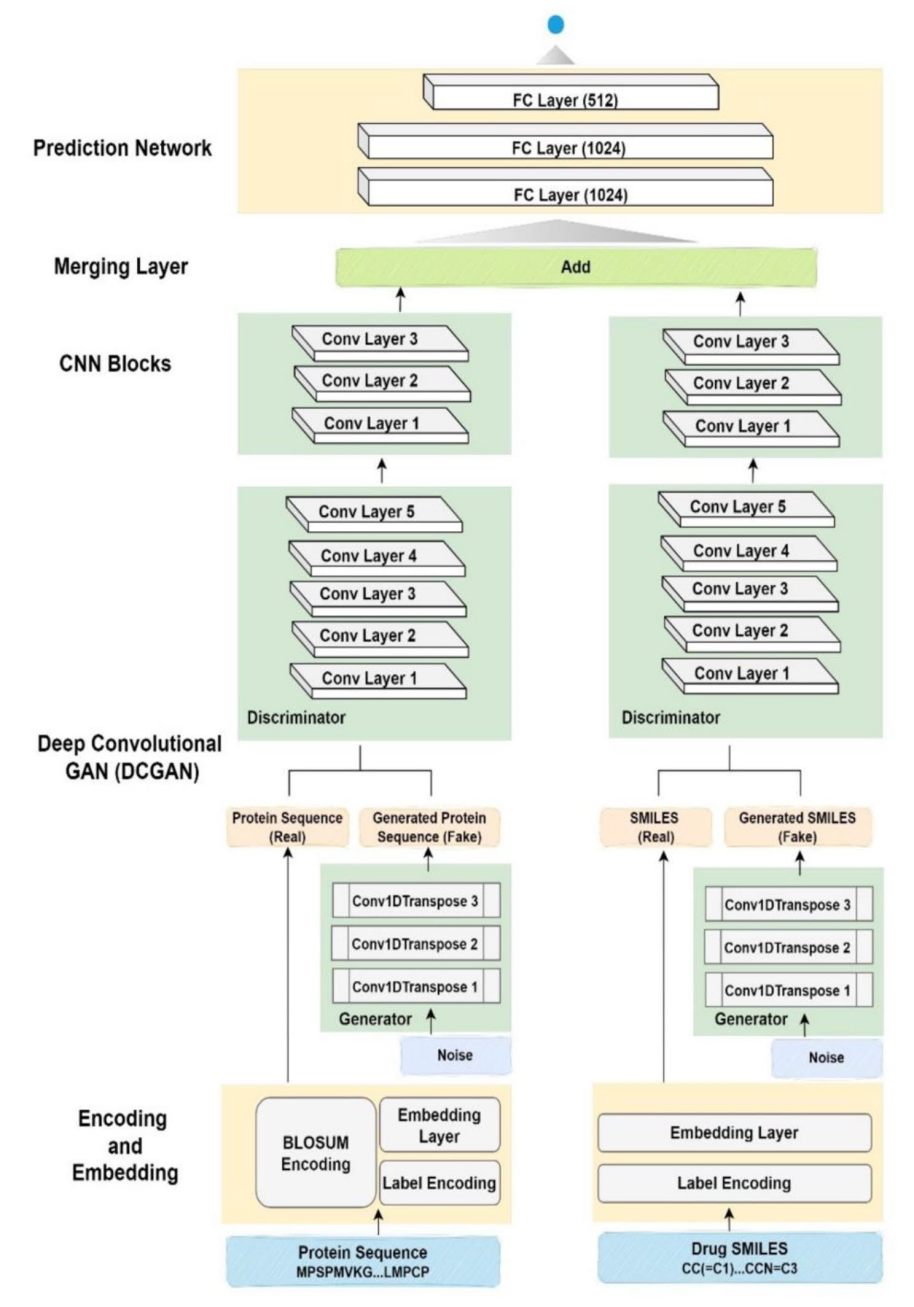
DCGAN-DTA method for DTA prediction. In the encoding and embedding step, the drug SMILES and protein sequences are encoded using a label encoding technique. The resulting encoded proteins and drugs are then embedded in the next step using an embedding layer. Additionally, to incorporate evolutionary features, the BLOSUM encoding technique is applied, transforming each amino acid in the protein sequences into a 25-dimensional feature vector. In the second step, two deep convolutional generative adversarial networks (DCGANs) are employed to provide representations for drug SMILES and protein sequences, along with CNN blocks. Both the generator and discriminator are trained through an adversarial process. The generator model generates fake protein sequences and drug SMILES, while the discriminator model distinguishes between real and fake sequences produced by the generator. The generator comprises three convolutional transpose layers for generating protein sequences and drug SMILES, while the discriminator consists of five CNN layers to discern the authenticity of sequences. In the third step, an add layer is utilized to merge the latent vectors for drugs and proteins. Finally, the last step includes a three-layer fully-connected block for drug-target interaction (DTA) prediction

DCGAN-DTA follows a four-step process for drug-target binding affinity (DTA) prediction, as illustrated in Fig. 1. The four steps include encoding and embedding, feature extraction, merging of latent vectors for drugs and proteins, and DTA prediction.

In the encoding and embedding step, drug SMILES and protein sequences undergo label encoding techniques where each character in the drug SMILES and protein sequence is converted into numerical data. This process results in the representation of the drug and protein as vectors with lengths equal to their corresponding SMILES and protein sequences. Padding is applied to ensure a fixed length for both the drug SMILES and protein sequences. The encoded proteins and drugs are then embedded in the next step using an embedding layer.

Furthermore, to incorporate evolutionary features, we utilized the BLOSUM encoding technique [43] for protein sequences. In this method, each amino acid in the protein sequences is converted into a 25-dimensional feature vector. As a result, the protein is represented as a matrix with dimensions of 25 multiplied by the length of the protein sequence.

In the second step, a customized version of deep convolutional generative adversarial networks (DCGANs) is employed to extract features from drug SMILES and protein sequences. The DCGAN consists of two models: the generator and the discriminator. These models are trained together using an adversarial process, where the generator generates fake protein sequences and drug SMILES, while the discriminator learns to distinguish between real and fake protein sequences and drug SMILES. Our DCGAN architecture diverges from the original DCGAN approach in several key aspects. Firstly, our model utilizes one-dimensional CNN (Conv1D) layers to effectively capture sequential patterns inherent in sequence data, including protein sequences and drug SMILES. This is in contrast to the two-dimensional CNN (Conv2D) layers typically used in traditional DCGAN architectures, which are more suited for processing spatial features in image data. Additionally, while the original DCGAN integrates Batch Normalization techniques within both the generator and discriminator networks, our implementation forgoes this approach due to observed performance degradation. We attribute this disparity to the nuanced nature of sequence data, where the application of Batch Normalization yielded suboptimal outcomes in our experimentation. Furthermore, in our implementation, the discriminator network employs Rectified Linear Unit (ReLU) activation functions to address the vanishing gradient problem and expedite convergence, which are particularly well-suited for sequence data (i.e. protein sequences and drug SMILES) dynamics. In contrast, the original DCGAN advocates for the use of Leaky ReLU activation functions in the discriminator, a choice optimized for image data processing tasks. The generator in DCGAN-DTA comprises three one-dimensional convolutional transpose (Conv1DTranspose) layers for generating protein sequences and drug SMILES. The first two layers employ the rectified linear unit (ReLU) activation function, while the final layer utilizes the hyperbolic tangent (tanh) activation function. The discriminator consists of five one-dimensional CNN (Conv1D) layers for discriminating between the fake and real sequences generated by the generator, each activated by the ReLU function. Subsequently, a flatten layer is employed, followed by a dense layer with a single node and a *tanh* activation function. DCGAN is trained using data collected from UniProt [44] and ChEMBL [45] databases, and the learned models are then utilized for the feature extraction step, along with CNN blocks consisting of three CNN layers and one max-pooling layer.

In the third step, we employed an add layer for merging latent vectors for drugs and proteins. The add layer, compared to a concatenation layer, provides a linear combination with a smaller size for the latent features learned from the representation learning step. Furthermore, based on various performed experiments, the performance of the model is improved with the add layer, compared to various merging layers, such as concatenation layer as employed in various DTA methods. The performance comparisons between the add layer utilized and the concatenation layer are presented in Section Results. Hence, the add layer provides smaller dimensions, leading to a reduced number of network parameters for the prediction task.

The final step involves a fully-connected block, which is commonly used in neural network-based methods for DTA prediction.

The training process was executed over 300 epochs, with a batch size of 256 for weight updates. Adam optimization algorithm was employed for training the networks, utilizing the learning rate of 0.001. In both datasets (BindingDB and PDBBind), protein sequences were set to a length of 2000, while SMILES representations of compounds were limited to a length of 200. The model utilized 128, 256, and 384 filters in different layers. The filter length for protein data was set to 8, while for drug data, it was set to 4. In the DCGAN architecture, the generator used 128, 64, and 1 filters, while the discriminator used 4, 8, 16, 32, and 64 filters. Both the generator and discriminator used a filter length of 3 in their convolutional layers. The number of neurons in the fully connected layers was set to 1024, 512, and 512. Additionally, a dropout rate of 0.25 was applied to prevent overfitting.

## Results

### Evaluation metrics

In our evaluation, we employed four commonly used performance metrics: the concordance index (CI), mean squared error (MSE), area under the precision-recall curve (AUPR), and ${r}_{m}^{2}$. The concordance index (CI) is a measure of prediction performance for a regression model. It is calculated using Eq. (1), where ${f}_{i}$ and ${f}_{j}$ represent the predicted values for the actual affinity values ${y}_{i}$ and ${y}_{j}$ (${y}_{i}>{y}_{j}$), respectively. In this equation, Z denotes a normalization constant. The function h is a step function defined by Eq. (2). The CI provides a measure of how well the model predicts the ordering of affinity values.

1 $$CI=\frac{1}{z}\sum _{{y}_{i}>{y}_{j}}h\left({f}_{i}-{f}_{j}\right)$$

2 $$h\left(x\right)=\left\{\begin{array}{c}1, if x>0\\ 0.5, if x=0\\ 0, if x<0\end{array}\right.$$

The second performance metric, mean squared error (MSE), is defined by Eq. (3). It quantifies the average squared difference between the predicted (P) and the ground-truth (Y) affinity values. Here, n is the number of samples. Lower MSE values indicate a closer match between the predicted and actual affinity values.

3 $$MSE=\frac{1}{n}\sum _{i=1}^{n}{\left({P}_{i}-{Y}_{i}\right)}^{2}$$

The third performance metric, the area under the precision-recall curve (AUPR), is used to evaluate the prediction performance of our method for a binary classification problem. In order to frame the classification problem, we transformed the binding affinities of both the PDBBind and BindingDB datasets into binary values. To accomplish this, we applied a threshold of 7 to the binding affinities.

The final widely-utilized metric, ${r}_{m}^{2}$​, characterizes the external prediction performance of a quantitative structure-activity relationship (QSAR) model. It is determined by Eq. (4), wherein ${r}^{2}$ and ${r}_{0}^{2}$ denote the squared correlation coefficients values with and without intercept, respectively [46–48]. Consequently, a model is deemed satisfactory if ${r}_{m}^{2}>0.5$.

4 $${r}_{m}^{2}= {r}^{2}\times (1-\sqrt{({r}^{2}-{r}_{0}^{2})})$$

### Implementation details

The development of our method was carried out using the Python programming language, utilizing the Keras and Tensorflow machine learning frameworks. The evaluation of our method on the benchmark datasets was conducted on a system running Ubuntu 18.04. The hardware specifications of the system include an Intel(R) Xeon(R) CPU @ 2.30 GHz and an NVIDIA GeForce GTX 1080 GPU with 11 GB of available memory. For training and evaluating the models, we employed a five-fold cross-validation approach. This involved dividing the dataset into five nearly equal-sized training and validation sets. The model was trained and hyperparameter tuning was performed using these sets. It is worth noting that the early stopping technique has been employed to prevent overfitting. The parameter settings for our model can be found in Supplementary Table 2.

### Comparisons for warm-start data splitting

To assess the prediction performance of our method, we compared it against seven alternative methods that employ different protein and drug representations. In selecting methods for comparison, we aimed to encompass a range of approaches in drug-target binding affinity prediction. We included established baseline methods including DeepDTA and GraphDTA for benchmarking, along with state-of-the-art techniques including FusionDTA, DGDTA, TEFDTA, and G-K BertDTA to assess novelty and potential advancements. Each method was chosen based on its relevance, availability of implementations or results, and practical considerations. Supplementary Table 3 presents additional information about these methods, including details about their approaches and the types of representations they utilize. As presented in Supplementary Table 3, we explored three distinct models named DCGAN-DTA (A), DCGAN-DTA (B), and DCGAN-DTA (C), which differ based on the encoding techniques employed and the use of DCGAN for protein representations.

**Fig. 2:**
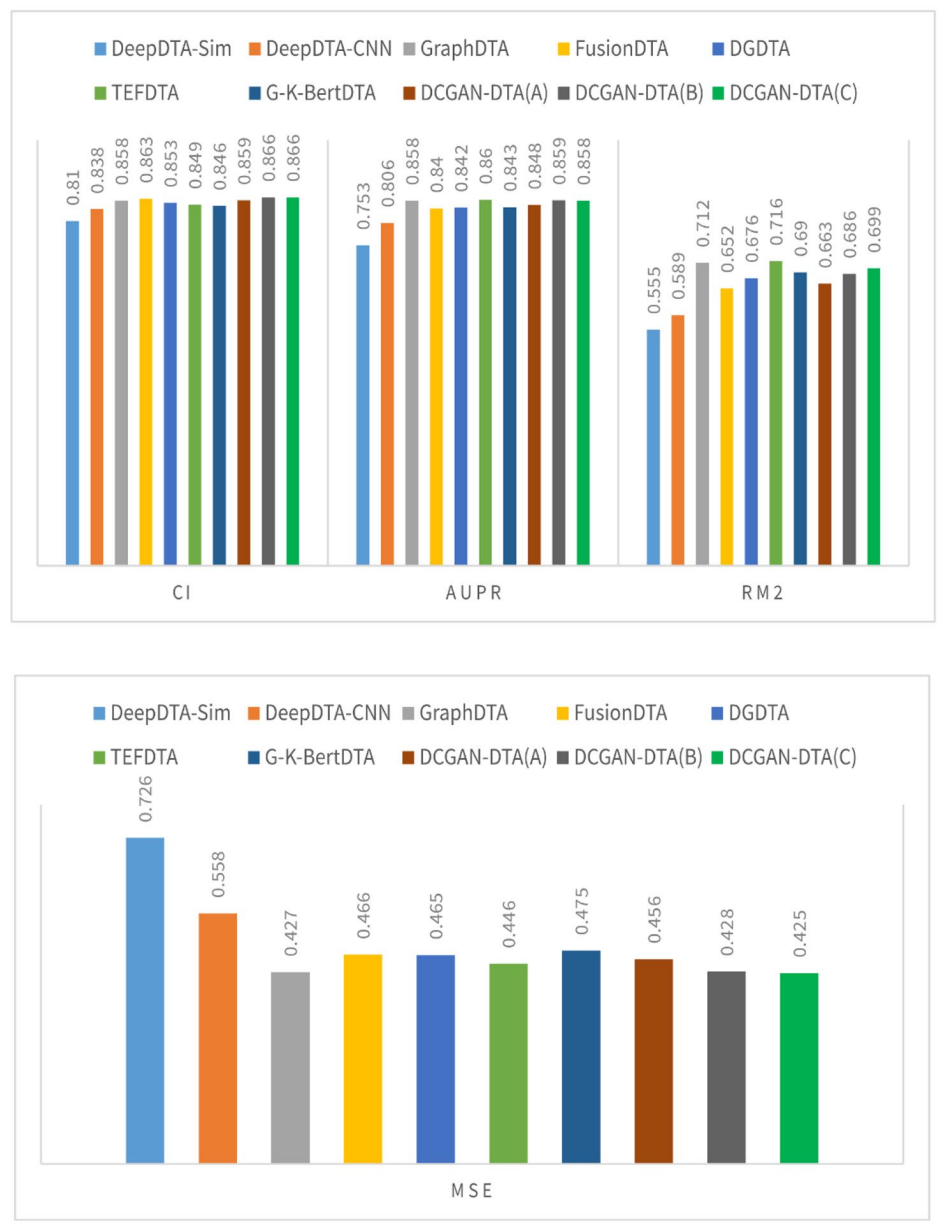
The CI, AUPR, ${\varvec{r}}_{\varvec{m}}^{2}$, and MSE for the DCGAN-DTA compared to the alternative methods – BindingDB dataset

**Fig. 3:**
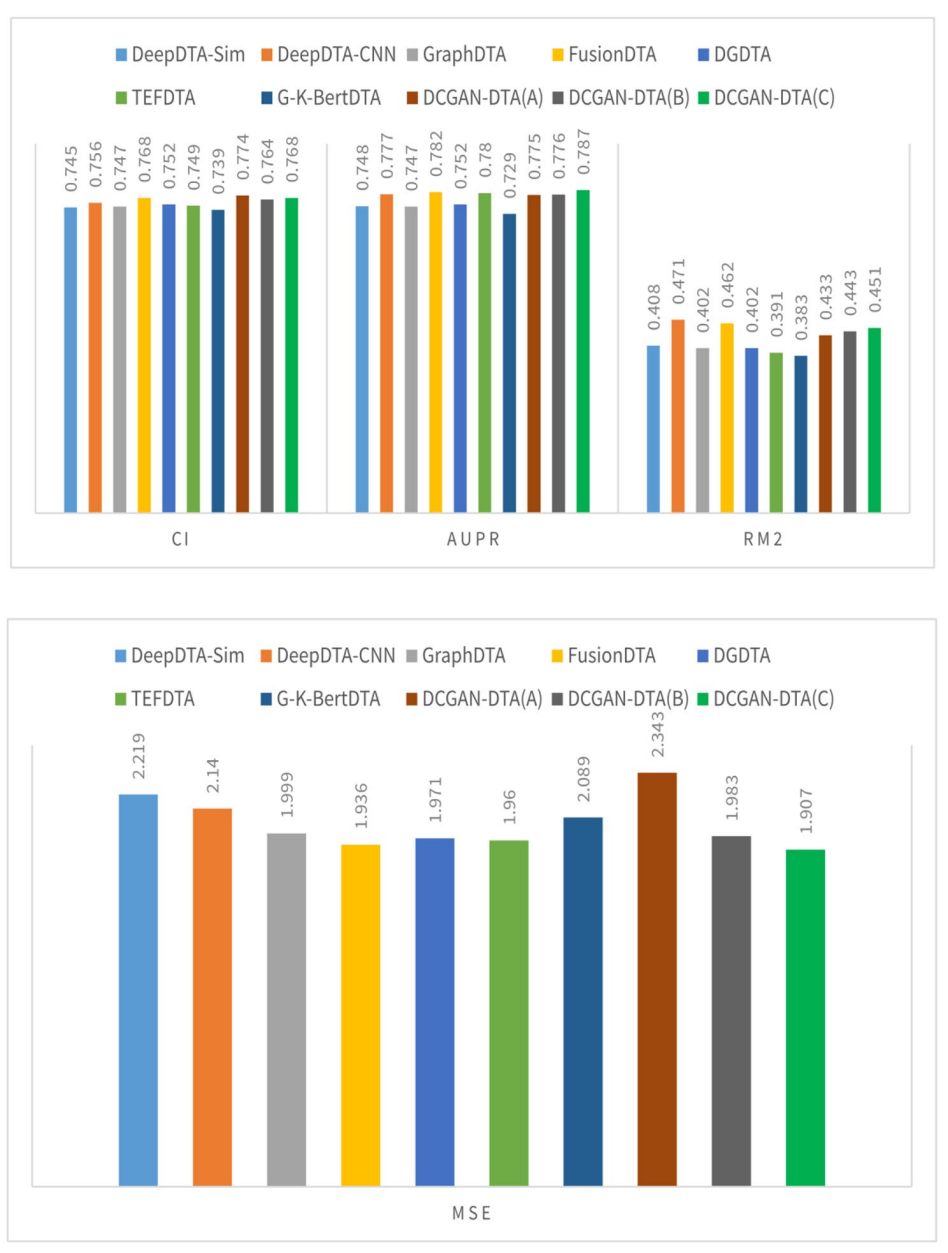
The CI, AUPR, ${\varvec{r}}_{\varvec{m}}^{2},$and MSE for the DCGAN-DTA compared to the alternative methods – PDBBind dataset

Figures 2 and 3 illustrate a comparison between three variations of DCGAN-DTA and alternative methods, using performance metrics including CI, MSE, AUPR, and${r}_{m}^{2}$. The evaluation was conducted on the BindingDB and PDBBind datasets, specifically for the warm-start data splitting setting.

According to Fig. 2, DCGAN-DTA exhibited superior performance compared to alternative methods in terms of CI and MSE, achieving the second-best AUPR, and third-best ${r}_{m}^{2}$ for the warm-start data splitting setting on the BindingDB dataset. Specifically, DCGAN-DTA (C) achieved the best CI and MSE, and the third-best ${r}_{m}^{2}$ while DCGAN-DTA (B) achieved the best CI and the second-best AUPR. In Fig. 3, DCGAN-DTA exhibited the best CI, AUPR, and MSE, and the third-best ${r}_{m}^{2}$ among the alternative methods, for the warm-start data splitting setting on the PDBBind dataset. Particularly, DCGAN-DTA (A) achieved the best CI, while DCGAN-DTA (C) demonstrated superior AUPR and MSE, and the third-best ${r}_{m}^{2}$.

Benchmark datasets vary in size, data distribution, and complexity, leading to variations in performance prediction results across different metrics. Based on Figs. 2 and 3, DCGAN-DTA consistently demonstrates prediction performance across all performance metrics for both DTA datasets. Specifically, DCGAN-DTA achieves the best CI for both datasets and the best AUPR for the larger dataset (i.e., BindingDB), as well as the second-best AUPR for the smaller dataset (i.e., PDBBind). Moreover, our method provides the third-best ${r}_{m}^{2}$ for both large and small datasets. While GraphDTA efficiently performs for all performance metrics on the BindingDB dataset, it does not provide efficient performance for the PDBBind dataset. Conversely, FusionDTA performs efficiently for the performance metrics on PDBBind, but its performance decreases for BindingDB. These findings suggest that the results for these alternative methods are sensitive to the distribution or ratio of available drug and protein data in DTA datasets.

The results for DCGAN-DTA (B) and (C), both of which employed BLOSUM encoding, demonstrate superior prediction performance in accordance with the data distribution displayed in Supplementary Fig. 1. Utilizing these models is most appropriate when dealing with protein sequences that have a wide distribution in terms of their sequence length. Moreover, based on the results for DCGAN-DTA (A), which demonstrated superior CI compared to the other versions for the PDBbind dataset, utilizing DCGAN for both protein and drug representations is more appropriate when the length of SMILES drugs is more widely distributed, and the length of protein sequences is less dispersed. Hence, each of the DCGAN-DTA models can be effectively employed for DTA prediction, accommodating the different distributions of drugs and targets within the dataset.

To further analyze the statistical significance of the results for predicting continuous binding affinity values, t-tests were performed between the methods with the first- and second-best CI scores. For a more comprehensive comparison, we included all three versions of DCGAN-DTA in the tests. Supplementary Fig. 2(a) and Fig. 2(b) illustrate the distribution of CI scores for DCGAN-DTA and FusionDTA in the warm-start data splitting setting for the BindingDB and PDBBind datasets, respectively. The *p*-value for each comparison is indicated on the plot using stars to denote the significance level of 95%. Based on Supplementary Fig. 2, the difference in CI between DCGAN-DTA and FusionDTA is statistically significant for both the BindingDB and PDBBind datasets. Specifically, the CI difference between DCGAN-DTA (C) and FusionDTA for the BindingDB dataset, as well as between DCGAN-DTA (A) and FusionDTA for the PDBBind dataset, is significant at a 95% confidence level.

**Fig. 4:**
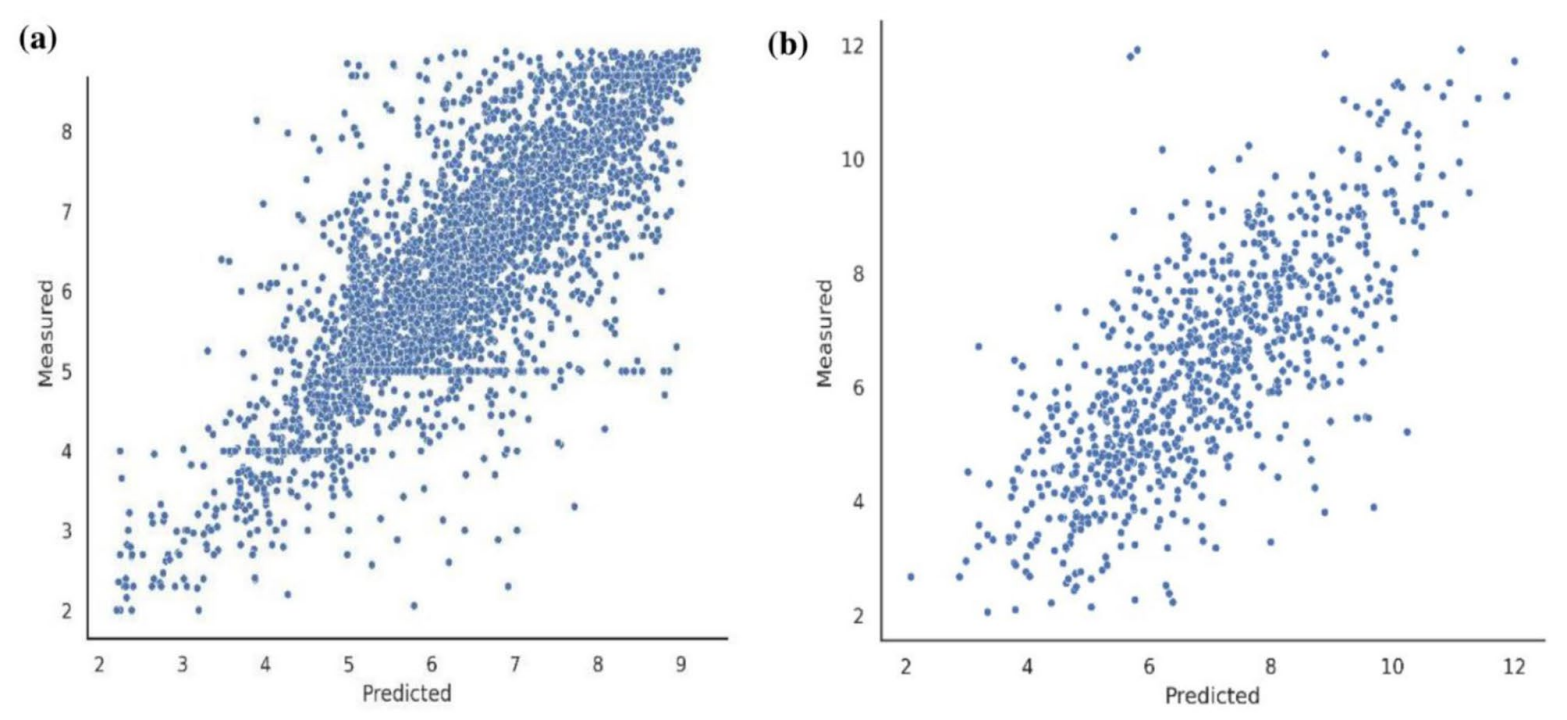
Predicted and actual binding affinities. (a) BindingDB, (b) PDBBind datasets

To assess the performance of DCGAN-DTA in predicting binding affinity values, we visually compared the proximity of the predicted values to the actual values, as depicted in Fig. 4. The figure illustrates that DCGAN-DTA successfully predicts binding affinity values that are in close agreement with the actual values for both the BindingDB and PDBBind datasets.

### Comparisons for cold-start drug data splitting

**Fig. 5:**
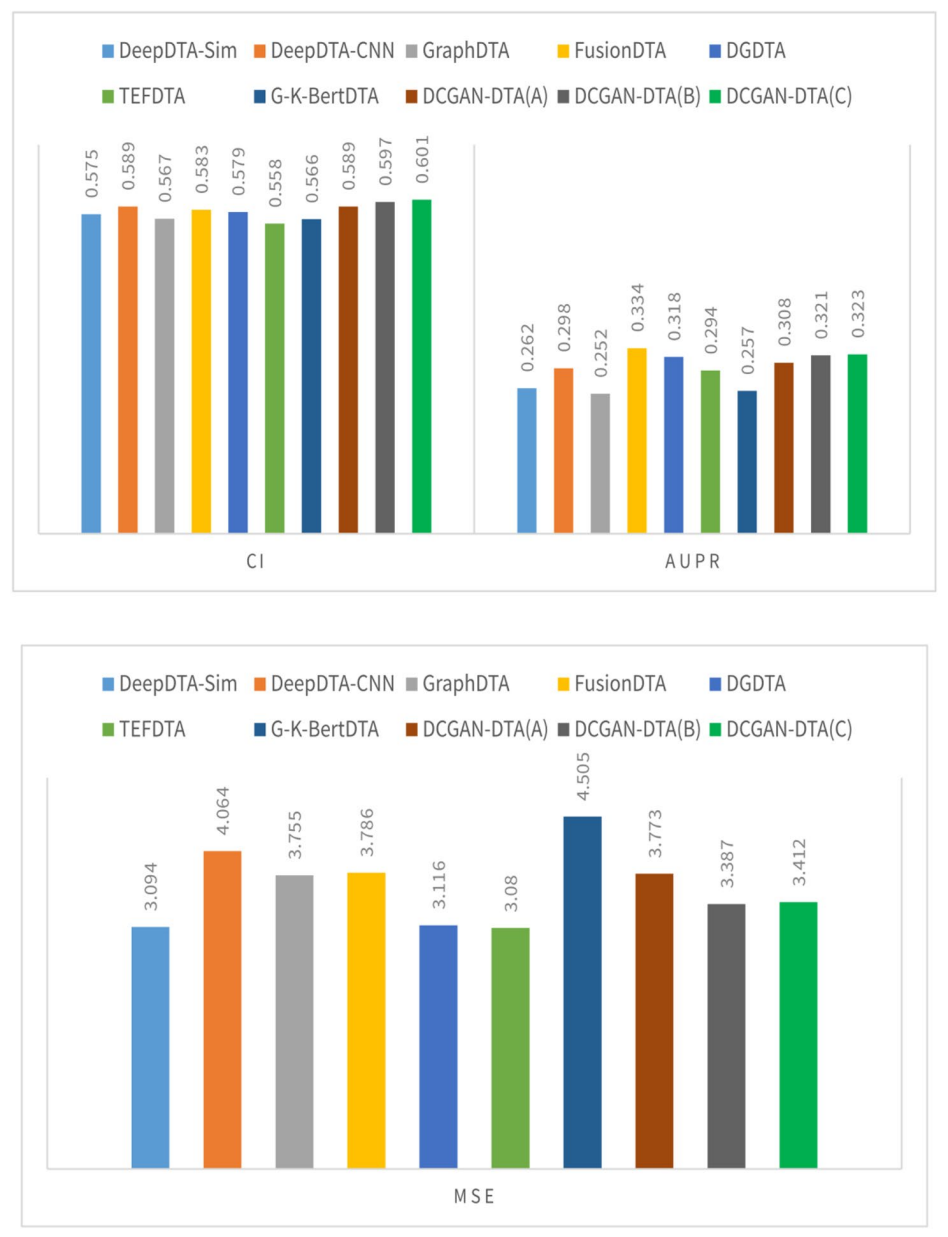
The CI, AUPR, and MSE for the DCGAN-DTA compared to the alternative methods for physiochemical properties splitting of PDBBind dataset based on open Babel logP

**Fig. 6:**
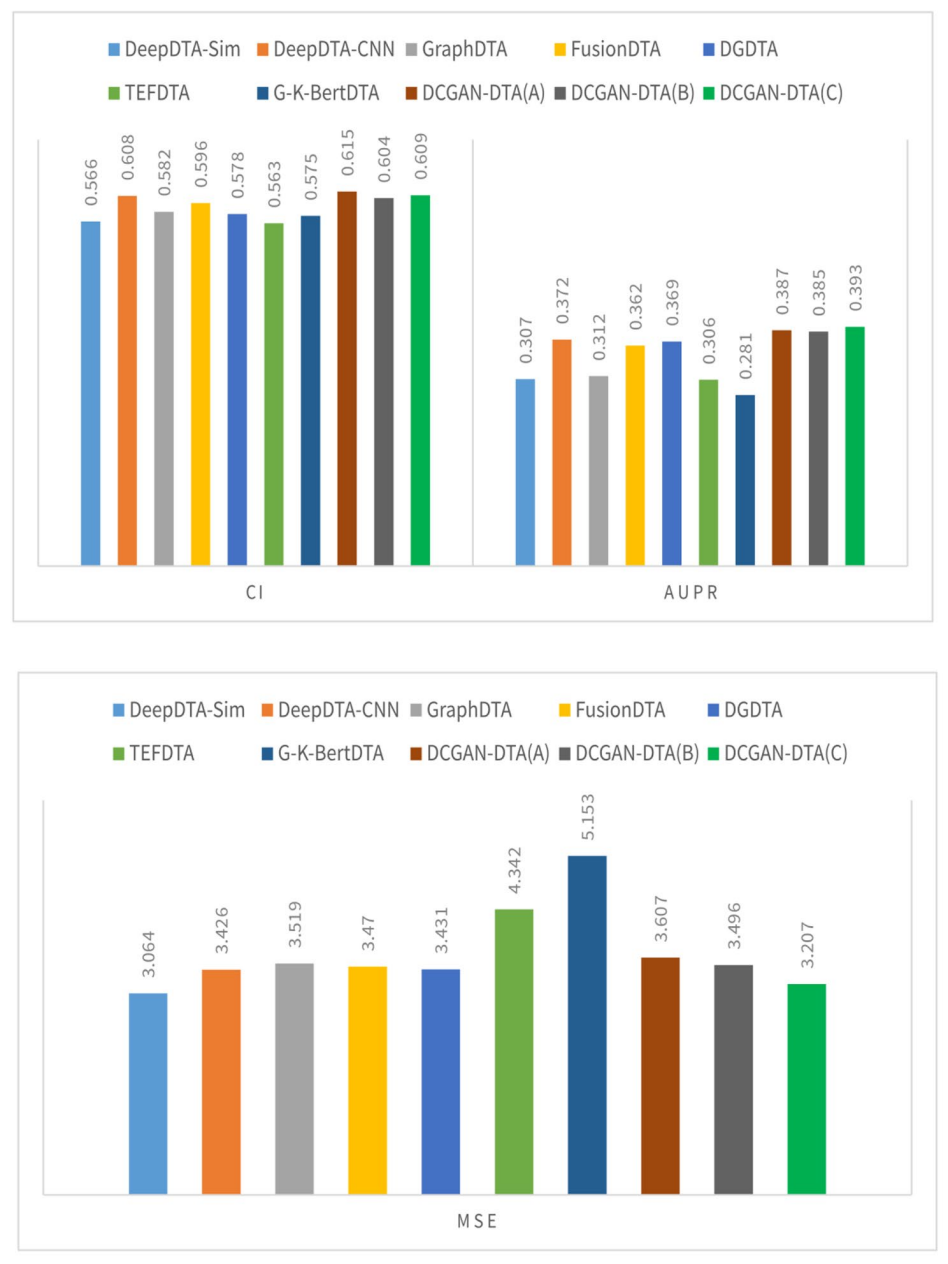
The CI, AUPR, and MSE for the DCGAN-DTA compared to the alternative methods for physiochemical properties splitting of PDBBind dataset based on XLOGP3

To assess the prediction performance of DCGAN-DTA for unseen drugs, we conducted two challenging cold-start data splitting settings based on the physiochemical properties of compound molecules. Specifically, we excluded the drug SMILES from the model training based on the logarithm of n-octanol-water partition coefficient (logP), which characterizes the lipophilicity of corresponding compounds. These excluded drugs were then utilized as the test data for evaluation. For this evaluation, we utilized the PDBBind dataset, which provides the necessary physiochemical information for the compounds, including logP values computed with Open Babel logP [49] and XLOGP3 [50] tools. Figures 5 and 6 present the comparison between DCGAN-DTA and alternative methods in terms of performance metrics, including CI, MSE, and AUPR, for the physiochemical properties splitting setting.

Based on Figs. 5 and 6, DCGAN-DTA showcased superior performance compared to alternative methods in terms of CI scores for both logP values, namely Open Babel logP and XLOGP3. Additionally, our method achieved the best AUPR for XLOGP3 and the second-best AUPR for Open Babel logP. Moreover, DCGAN-DTA achieved the second-best MSE for XLOGP3 data splitting settings. While DeepDTA efficiently performs in terms of CI (i.e., second-best CI) for Open Babel logP and XLOGP3, its performance decreases, particularly for Open Babel logP, when considering other performance metrics. These findings suggest that the results for this alternative method are sensitive to the distribution or ratio of available drug and protein data in DTA datasets. Overall, DCGAN-DTA exhibited superior performance in predicting binding affinity values and interactions for unseen data splitting based on molecular properties.

To assess the statistical significance of the results for binding affinity prediction, we conducted t-tests between the methods that achieved the first- and second-best CI scores. For a more comprehensive comparison, we included all three versions of DCGAN-DTA in the tests. In Supplementary Fig. 3 (a, b), the distribution of CI scores for DCGAN-DTA and DeepDTA-CNN is presented for the physiochemical properties data splitting setting using the PDBBind dataset. The *p*-value for each comparison is indicated on the plot using stars to represent the 95% significance level. The annotation ‘ns’ indicates that the difference in results between those methods is not statistically significant. Based on Supplementary Fig. 3, the statistical tests suggest that DCGAN-DTA significantly outperforms DeepDTA-CNN in terms of CI for data splitting based on Open Babel logP. However, the CI difference between DeepDTA-CNN and DCGAN-DTA is not statistically significant for the XLOGP3 data splitting setting.

### Adversarial control experiments

**Fig. 7:**
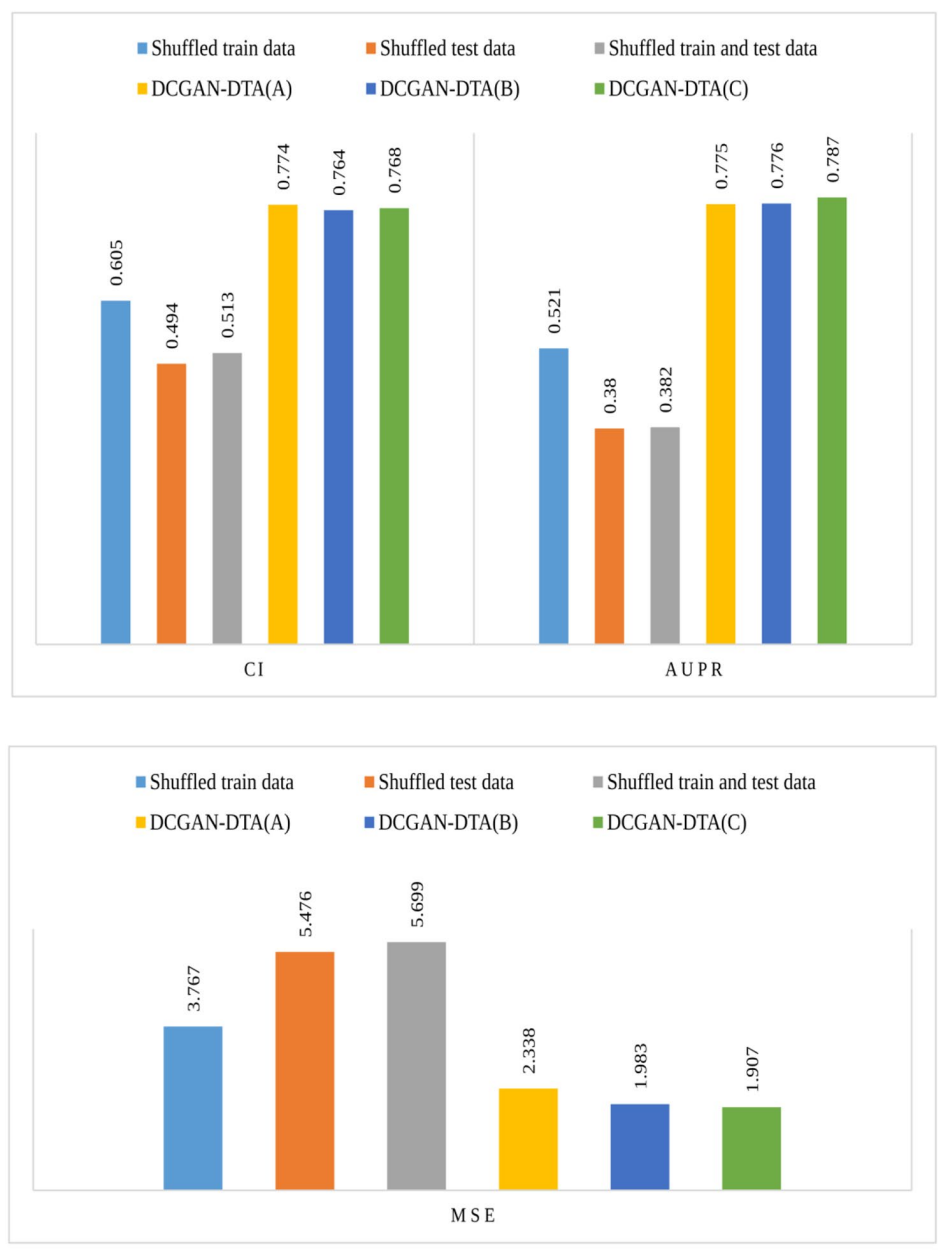
The CI, AUPR and MSE for the DCGAN-DTA on shuffled binding affinities for train, test, and train and test data for PDBBind dataset

To verify the robustness of the prediction performance of DCGAN-DTA, we conducted multiple adversarial control experiments. Firstly, we evaluated the method using straw models that were trained and tested on shuffled binding affinity values. We performed three different experiments: training models using shuffled data and testing them on actual data, training models using actual data and testing them on shuffled data, and training and testing models using shuffled data. Figure 7 illustrates the prediction performance of DCGAN-DTA on shuffled binding affinity values using three performance metrics: CI, AUPR, and MSE. It should be noted that the experiments on shuffled data were specifically performed on DCGAN-DTA (C). Additionally, for comprehensive comparisons, we included the predictive performance of all three models of DCGAN-DTA, namely DCGAN-DTA (A), (B), and (C).

As shown in Fig. 7, all experiments on straw models result in a loss of predictive performance across the three performance metrics. Therefore, we can conclude that our method is robust against confounding variables and data artifacts.

We conducted t-tests to assess the statistical significance of the results for binding affinity value prediction, comparing the methods based on the CI metric. Supplementary Fig. 4 displays the distribution of CI scores for DCGAN-DTA across both unshuffled data and three shuffled data settings. The *p*-value for each comparison is indicated on the plot using stars, representing the level of significance. As shown in Supplementary Fig. 4, DCGAN-DTA exhibited statistically significant prediction performance at a significance level greater than 95% for all three comparisons.

**Fig. 8:**
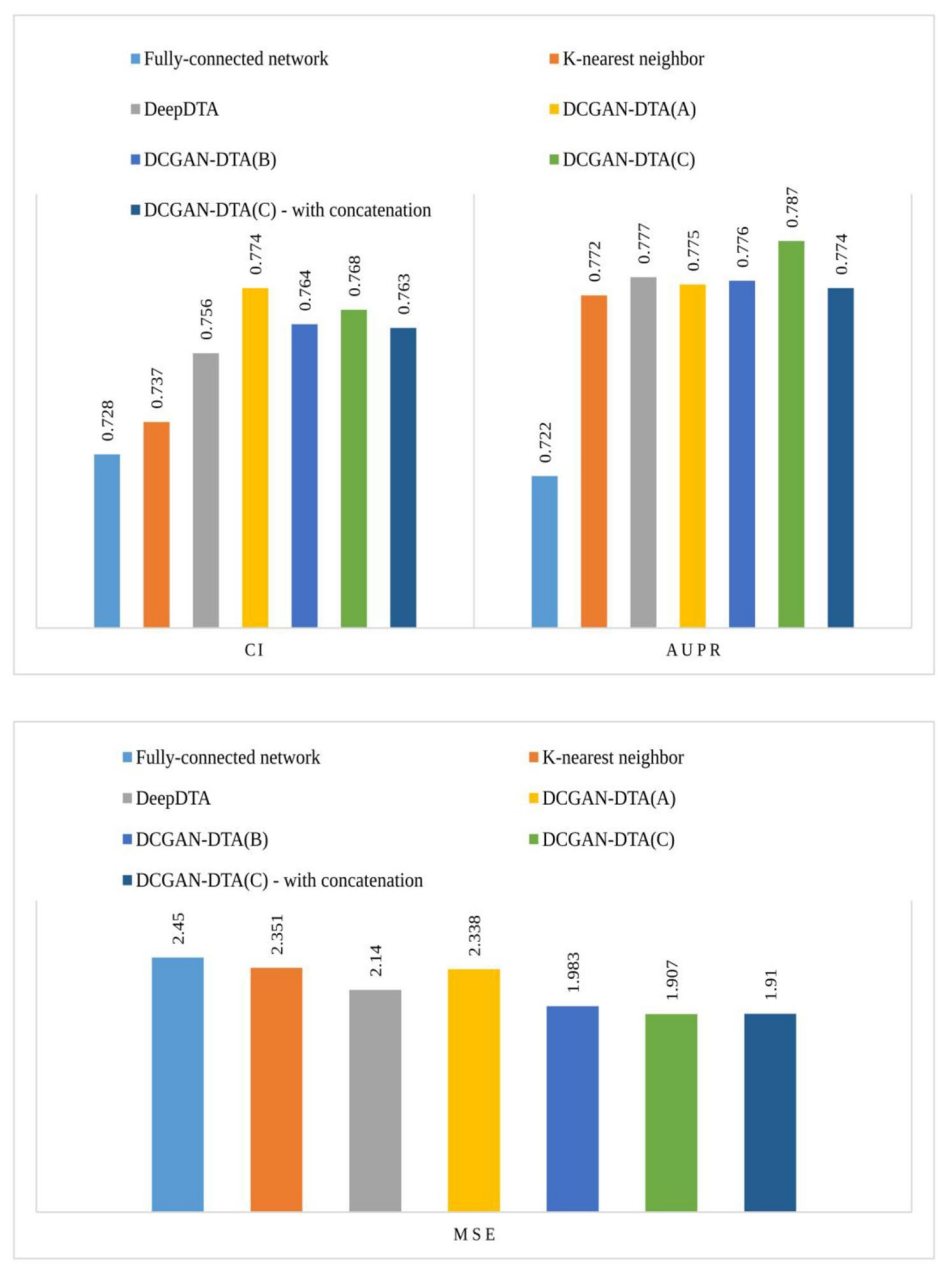
The CI, AUPR and MSE for the DCGAN-DTA and lightweight networks for PDBBind dataset

In addition, we compared the prediction performance of DCGAN-DTA against two baselines: a fully-connected network and k-nearest neighbor models trained using the PDBBind dataset encoded using the label encoding technique. Additionally, for a more comprehensive analysis, we included DeepDTA, which utilizes the DCGAN for neither protein nor drugs. Figure 8 illustrates the prediction performance of our method compared to the baselines for the PDBBind dataset across the three performance metrics. As shown in the figure, DCGAN-DTA outperformed the baselines, achieving the best CI, MSE, and AUPR scores.

Furthermore, we investigated the utilization of different layers for merging the latent vectors learned in the representation learning step. To achieve this, we conducted experiments using both the add layer employed in DCGAN-DTA and the widely-used concatenation layer for DTA prediction. The performance comparisons for these two layers in the DCGAN-DTA network are presented in Fig. 8.

As shown in Fig. 8, DCGAN-DTA with the add layer demonstrated superior prediction performance compared to DCGAN-DTA with the concatenation layer across all three performance metrics.

## Discussion

The prediction of binding affinity and interactions between drugs and proteins plays a crucial role in drug development and drug repurposing for disease treatment. However, machine learning-based methods for drug-target interaction (DTA) face several challenges, such as limited labeled data availability for learning drug and protein representations, as well as reliance on limited validation strategies. To address these drawbacks, we propose DCGAN-DTA. It is a method for DTA prediction that leverages a convolutional neural network (CNN)-based generative adversarial network (GAN) to learn representations of drug SMILES and protein sequences. We also employ multiple validation strategies to evaluate the effectiveness of our method in real-life DTA prediction scenarios.

DCGAN-DTA overcomes limitations by utilizing a DCGAN for representation learning of drug SMILES and protein sequences. We evaluate the method using warm-start and cold-start physiochemical-based data splitting settings, which provide comprehensive assessments. Additionally, we conduct multiple adversarial control experiments using straw models to demonstrate the robustness and generalization of our method’s prediction utility.

We compare DCGAN-DTA against alternative methods in four groups: similarity-based, sequence-based, graph-based, and transformer-based methods for DTA prediction. Compared to similarity-based methods, our approach utilizes raw protein sequences and drug SMILES without feature selection or engineering, thus eliminating the need for constructing multiple similarity matrices. By leveraging deep neural networks, DCGAN-DTA enables automatic feature extraction to capture complex and non-linear features, resulting in superior prediction performance.

Compared to sequence-based methods like DeepDTA, DCGAN-DTA employs CNN-based GANs trained on a larger amount of available unlabeled data. Protein sequences are encoded using BLOSUM encoding matrices, allowing us to capture evolutionary relationships. Additionally, DCGANs utilize an add layer instead of the commonly used concatenation layer for merging latent vectors of proteins and drugs in the final prediction network.

In contrast to graph-based methods, which rely on graph modeling of compound and protein molecules, our method learns from raw sequence data obtained from available databases. Compared to GraphDTA, and DGDTA, DCGAN-DTA employs deep convolutional GANs for both drug and protein representation learning. Furthermore, we use BLOSUM-encoded protein sequences to incorporate evolutionary features in the feature extraction and prediction steps. Consequently, our method can make predictions based on simple yet rich encoded data, without the need for additional graphical data modeling.

We also compare our method against transformer-based methods including Fusion-DTA, TEFDTA, and G-K BertDTA. In contrast to transformer-based approaches, DCGAN-DTA leverages deep convolutional GANs for representation learning from protein sequences and drug SMILES. Besides demonstrating superior predictive performance in various experiments presented in Section Results, DCGAN-DTA relies on CNN layers for both drugs and targets, which helps prevent computational overhead in terms of time and space complexity.

Overall, DCGAN-DTA demonstrates superior DTA prediction performance compared to baselines and some state-of-the-art methods across various data splitting settings. By leveraging the DCGAN architecture, which provides distributed representations for drugs and proteins, utilizing more unlabeled data for training, and employing efficient protein sequence encoding that captures evolutionary information, our method achieves strong predictive performance for DTA. Consequently, DCGAN-DTA can be employed for DTA prediction based on protein sequences and drug SMILES, accelerating drug development and repurposing processes.

Furthermore, our approach can be extended to address similar biological challenges, such as predicting associations between miRNAs and small molecules, which involve sequence data. In recent years, there has been a growing identification of non-coding RNAs (ncRNAs), with mounting evidence suggesting their potential impact on gene expression and disease progression [51–53]. This emerging understanding positions ncRNAs, particularly miRNAs, as a promising class of drug targets [51–53]. Developing methods that utilize generative models to provide predictions based on biological sequence data, or customizing the proposed method with modifications, can be considered as a future work to effectively predict associations between small molecules and miRNAs.

## Conclusion

In this study, we introduced DCGAN-DTA, a novel approach for predicting drug-target binding affinity. Our proposed method leverages deep convolutional generative adversarial networks (DCGANs) to learn representations of both proteins and drugs. Additionally, DCGAN-DTA utilizes evolutionary features for proteins through the BLOSUM encoding technique. Compared to various existing methods for predicting drug-target binding affinity, DCGAN-DTA demonstrates superior performance across multiple validation strategies. To underscore its reliability, we further evaluated DCGAN-DTA through multiple adversarial control experiments. The findings of this study underscore the potential of DCGAN-DTA to expedite drug repurposing efforts, facilitate novel drug discovery, and ultimately enhance disease treatment.

In addition to its efficient and robust performance in drug-target affinity (DTA) prediction, DCGAN-DTA offers data efficiency for training on small datasets. Specifically, when access to labeled data is limited, leveraging DCGAN-DTA can prove efficient due to its capability to extract hierarchical representations of data features from unlabeled data, facilitated by the convolutional neural networks (CNNs) architecture used in DCGANs. This enables the proposed method to provide efficient predictions even with small labeled datasets for DTA prediction. Moreover, the transferability and reusability of the model for similar biological problems are key features of DCGAN-DTA. By training DCGANs on large databases of protein sequences and drug SMILES, each trained DCGAN can be fine-tuned for other bioinformatics and drug discovery problems that require efficient learning of protein and drug representations.

With the increasing availability of data for Drug-Target Affinity (DTA) prediction and advancements in GPU architectures, one potential future direction for DTA prediction could involve the utilization of new generative modeling techniques, such as diffusion models. Furthermore, large language models and transformers, which have shown promising efficacy in processing protein sequence data, can enhance DTA prediction. On the other hand, graph modeling and the utilization of advanced Graph Neural Networks (GNNs) for drugs can improve molecular modeling and enhance DTA prediction.

### Electronic supplementary material

Below is the link to the electronic supplementary material.

Supplementary Material 1

Supplementary Material 2

Supplementary Material 3

Supplementary Material 4

Supplementary Material 5

Supplementary Material 6

Supplementary Material 7

## Data Availability

The datasets generated and/or analyzed during the current study are available in the GitHub repository, https://github.com/mojtabaze7/DCGAN-DTA. The DCGAN-DTA web server is accessible at https://dcgan.shinyapps.io/bindingaffinity/.
