# Supplementary material for: DCGAN-DTA: Predicting drug-target binding affinity with deep convolutional generative adversarial networks
Source: BMC Genomics. 2024 May 9;25:411. doi: 10.1186/s12864-024-10326-x (PMC11080241; doi:10.1186/s12864-024-10326-x)
Supplement: Supplementary file 1 — Supplementary Material 1 [file 12864_2024_10326_MOESM1_ESM.docx]

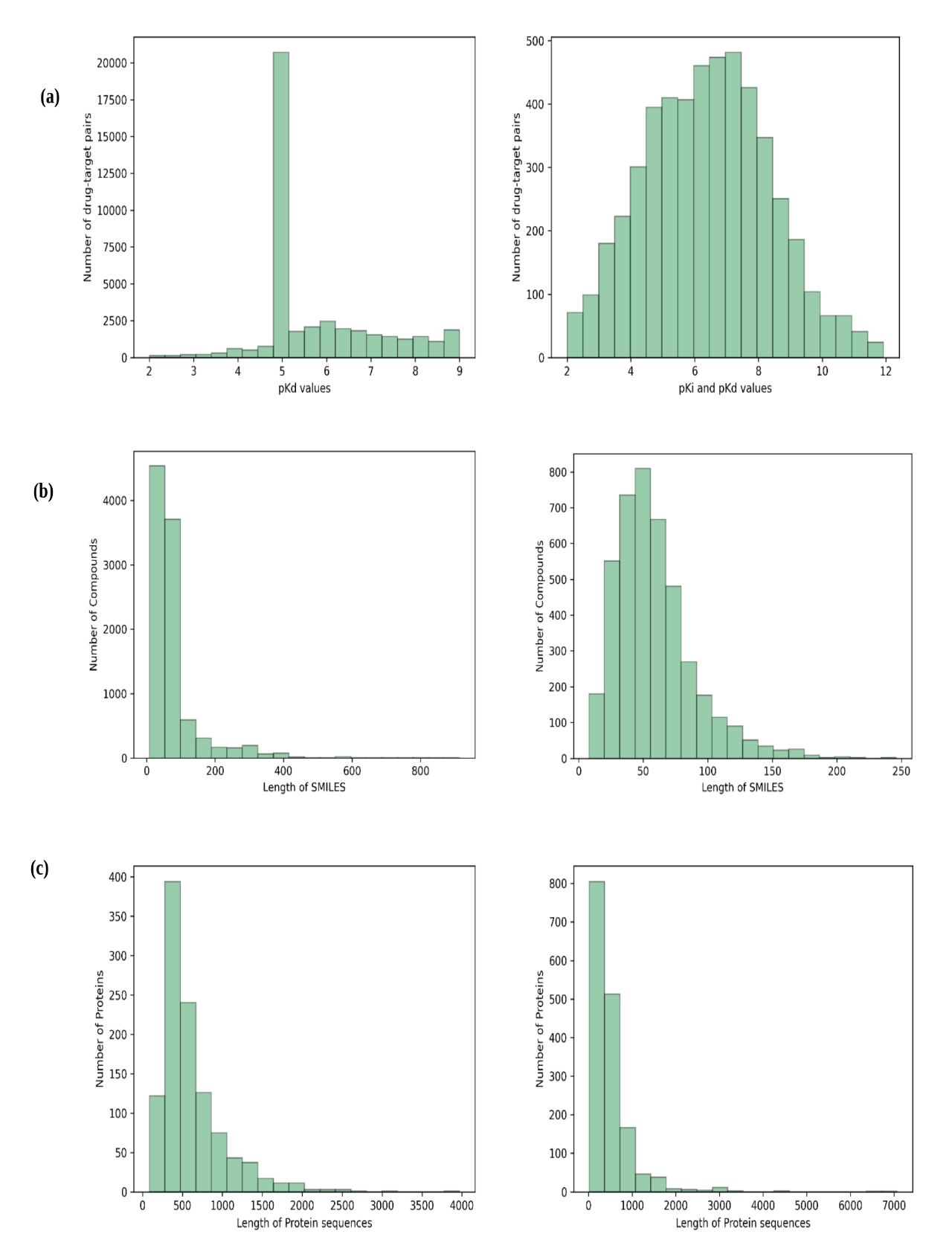


**Supplementary Fig. 1 More details for benchmark datasets. (a)** Affinity values distribution (the pKd values for BindingDB and the pKi, and pKd values for PDBbind datasets) and **(b)** the lengths of the drug in SMILES format and **(c)** the protein sequences for BindingDB and PDBbind datasets
