# Supplementary material for: DCGAN-DTA: Predicting drug-target binding affinity with deep convolutional generative adversarial networks
Source: BMC Genomics. 2024 May 9;25:411. doi: 10.1186/s12864-024-10326-x (PMC11080241; doi:10.1186/s12864-024-10326-x)
Supplement: Supplementary file 3 — Supplementary Material 3 [file 12864_2024_10326_MOESM3_ESM.docx]

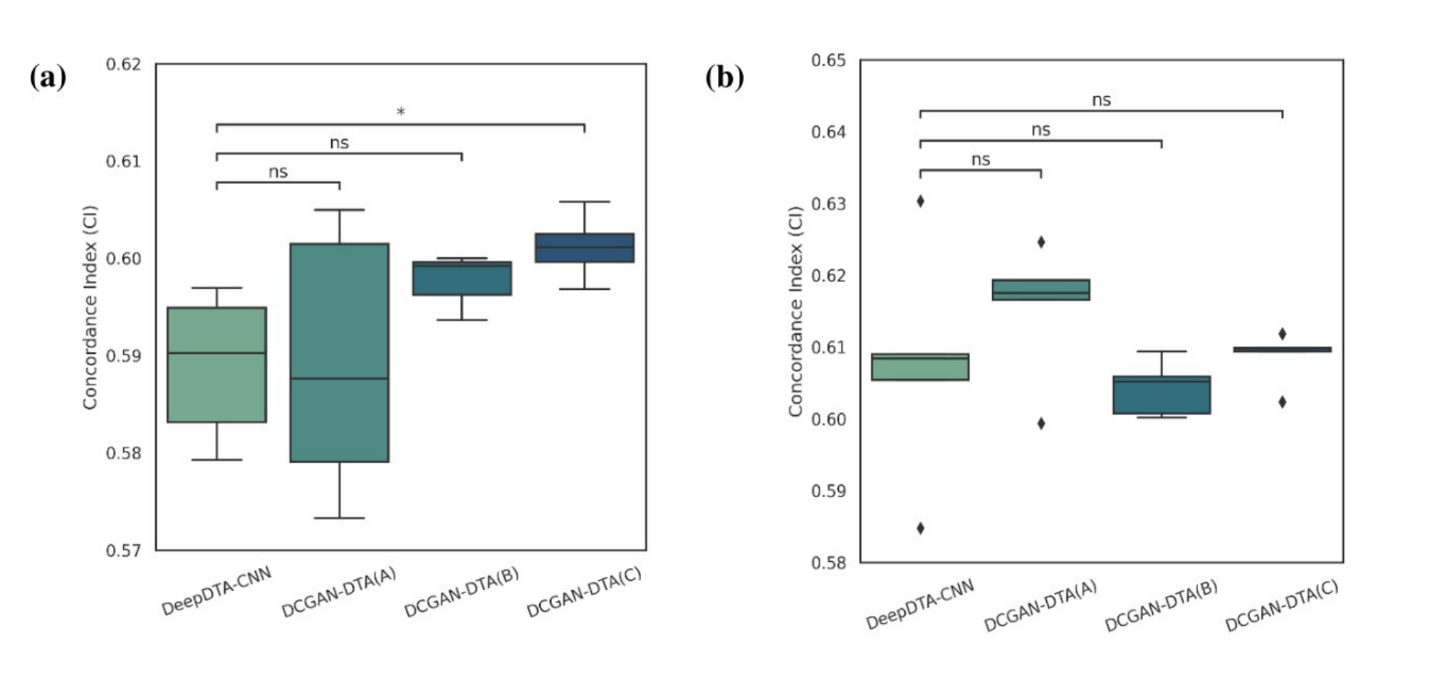


**Supplementary Fig. 3 The distribution of CI scores for the two best methods for cold-drug data splitting setting** for PDBBind**.** (a) Open Babel logP, (b) XLOGP3. For a more comprehensive comparison, we included all three versions of DCGAN-DTA in the tests.
