# Supplementary material for: DCGAN-DTA: Predicting drug-target binding affinity with deep convolutional generative adversarial networks
Source: BMC Genomics. 2024 May 9;25:411. doi: 10.1186/s12864-024-10326-x (PMC11080241; doi:10.1186/s12864-024-10326-x)
Supplement: Supplementary file 4 — Supplementary Material 4 [file 12864_2024_10326_MOESM4_ESM.docx]

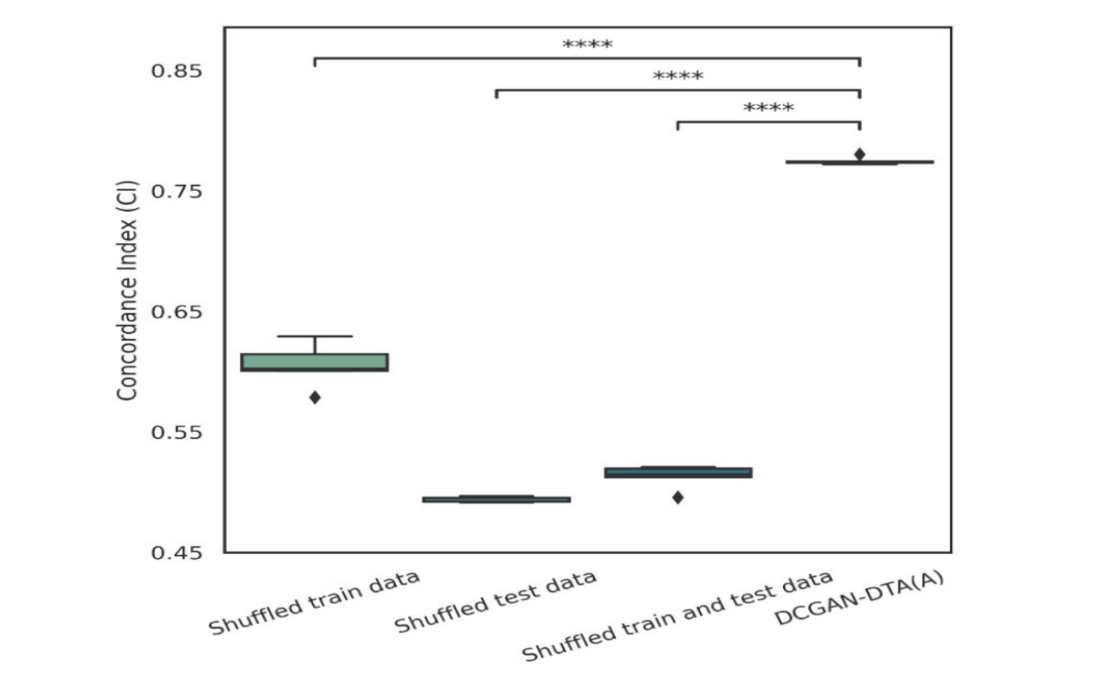


**Supplementary Fig. 4 The distribution of CI scores for adversarial control experiments for DCGAN-DTA and three shuffled data setting**
