# Supplementary material for: DCGAN-DTA: Predicting drug-target binding affinity with deep convolutional generative adversarial networks
Source: BMC Genomics. 2024 May 9;25:411. doi: 10.1186/s12864-024-10326-x (PMC11080241; doi:10.1186/s12864-024-10326-x)
Supplement: Supplementary file 5 — Supplementary Material 5 [file 12864_2024_10326_MOESM5_ESM.docx]

**Supplementary Table 1 The summary of BindingDB and PDBBind datasets**

|  | Proteins | Compounds | Interactions | Affinity measure |
| --- | --- | --- | --- | --- |
| BindingDB | 1088 | 9864 | 42203 | Dissociation constant (Kd) |
| PDBbind | 1606 | 4231 | 5014 | Inhibition constant (Ki)  Dissociation constant (Kd) |
