# Supplementary material for: DCGAN-DTA: Predicting drug-target binding affinity with deep convolutional generative adversarial networks
Source: BMC Genomics. 2024 May 9;25:411. doi: 10.1186/s12864-024-10326-x (PMC11080241; doi:10.1186/s12864-024-10326-x)
Supplement: Supplementary file 6 — Supplementary Material 6 [file 12864_2024_10326_MOESM6_ESM.docx]

**Supplementary Table 2 parameters settings for our models**

| Length of protein sequences | 2000 |
| --- | --- |
| Length of SMILES | 200 |
| Number of filters | 128,256,384 |
| Filter length (protein) | 8 |
| Filter length (drug) | 4 |
| DCGAN number of filters (Generator) | 128.64,1 |
| DCGAN number of filters (Discriminator) | 4,8,16,32,64 |
| DCGAN Filter length (Generator) | 3 |
| DCGAN Filter length (Discriminator) | 3 |
| Number of neurons | 1024,512,512 |
| Number of epochs | 300 |
| Batch size | 256 |
| Optimizer function | adam |
| Dropout | 0.25 |
| Learning rate | 0.001 |
