# Supplementary material for: DCGAN-DTA: Predicting drug-target binding affinity with deep convolutional generative adversarial networks
Source: BMC Genomics. 2024 May 9;25:411. doi: 10.1186/s12864-024-10326-x (PMC11080241; doi:10.1186/s12864-024-10326-x)
Supplement: Supplementary file 7 — Supplementary Material 7 [file 12864_2024_10326_MOESM7_ESM.docx]

**Supplementary Table 3 Alternative methods for comparison studies and their protein and drug representations**

|  | Proteins | Drugs |
| --- | --- | --- |
| DeepDTA-Sim | Smith-Waterman similarities | Label encoding, embedding layer ,and 1DCNN |
| DeepDTA-CNN | Label encoding, embedding layer ,and 1DCNN | Label encoding, embedding layer ,and 1DCNN |
| GraphDTA | Label encoding, embedding layer ,and 1DCNN | Graph neural networks(GNNs) |
| FusionDTA | Transformers, Feed-Forward networks, and BiLSTM | Feed-Forward networks, and BiLSTM |
| DGDTA | Bi-LSTM, and CNN | Dynamic graph attention networks |
| TEFDTA | Label encoding, embedding layer ,and 1DCNN | MACCS fingerprint, embedding layer, and Transformer |
| G-K BertDTA | CNN, and DenseSENet (DenseNet and squeeze-and-excitation (SE) blocks) | Graph isomorphism network (GIN), and KB-BERT |
| DCGAN-DTA(A) | Label encoding, embedding layer, DCGAN, and 1DCNN | Label encoding, embedding layer, DCGAN, and 1DCNN |
| DCGAN-DTA(B) | BLOSUM encoding, DCGAN, and 1DCNN |  |
| DCGAN-DTA(C) | BLOSUM encoding, and 1DCNN |  |
